# The Effect of Seasonal Priming on Specific Inhalation Challenges With Birch and Grass Allergen Among Persons With Allergic Rhinitis

**DOI:** 10.3389/falgy.2021.737799

**Published:** 2021-10-21

**Authors:** Pia V. Ørby, Jakob H. Bønløkke, Bo M. Bibby, Peter Ravn, Ole Hertel, Torben Sigsgaard, Vivi Schlünssen

**Affiliations:** ^1^Department of Public Health, Environment, Work and Health, Danish Ramazzini Centre, Aarhus University, Aarhus, Denmark; ^2^Department of Environmental Science, Aarhus University, Roskilde, Denmark; ^3^Danish Big Data Centre for Environment and Health (BERTHA), Aarhus University, Roskilde, Denmark; ^4^Department of Occupational and Environmental Medicine, Danish Ramazzini Centre, Aalborg University Hospital, Aalborg, Denmark; ^5^Department of Ecoscience, Aarhus University, Roskilde, Denmark; ^6^National Research Centre for the Working Environment, Copenhagen, Denmark

**Keywords:** pollen allergy, allergic rhinitis, allergic asthma, seasonal priming, outdoor occupations, PD_20_, bronchial response, specific inhalation challenge

## Abstract

**Objectives:** Allergic diseases are prevalent in the working population, and work-related airborne pollen exposure might be substantial, especially among outdoor workers, resulting in work-exacerbated effects. Seasonal exposure to pollen may induce a priming effect on the allergic bronchial response resulting in exaggerated effects at the end of the natural pollen season. This was previously observed among people with asthma but may also be of importance for persons with allergic rhinitis. In this study, we examined the effect of seasonal priming on bronchial responsiveness among young adults with allergic rhinitis and no or mild asthma. In addition, we explored the association between the baseline characteristics of participants and the severity of bronchoconstriction. Finally, we evaluated the application of a novel non-linear regression model to the log-dose-response curves.

**Material and methods:** In a crossover design, 36 participants underwent specific inhalation challenges (SICs) with either grass or birch allergen outside and at the end of the pollen season. The differences in bronchial response were evaluated by comparing the dose-response profiles and PD_20_ estimates derived by applying a non-linear regression model.

**Results:** The results showed that 12 of the 19 grass pollen-exposed participants had a lower PD_20_ at the end of the season compared with the outside season. For birch, this was true for nine out of the 17 participants. However, no statistically significant effects of the seasonal pollen exposure were found on neither the shape nor the magnitude of the modeled dose-response curves for either birch allergen, *p* = 0.77, or grass allergen, *p* = 0.45. The model depicted a good fit for the data. Among the baseline characteristics, only the size of the skin prick test for grass allergen was associated with PD_20_.

**Conclusion:** This study does not support a priming effect of pollen exposure on the bronchial response from the natural seasonal exposure levels of grass or birch allergens among young adults with allergic rhinitis.

## Article Summary

### Article Focus

– To study the priming effect of seasonal pollen exposure, relevant for outdoor occupations, on the bronchial responsiveness of persons with allergic rhinitis using a specific inhalation challenge (SIC) test.

### Key Messages

– This study does not support a priming effect of seasonal exposure of pollen on the bronchial response of persons with allergic rhinitis.
– Increased size of skin prick test for grass predicted stronger bronchial responsiveness for grass allergen.
– Future studies of the priming effect of seasonal exposure should focus on the impact of IgE-levels and the response patterns.

### Strengths and Limitations of This Study

– This is the first study to comprehensively examine the effect of the priming effect of seasonal pollen exposure on bronchial responsiveness using the SIC test on persons with allergic rhinitis.
– The application of a four-parameter model for PD_20_-estimates presents a new approach that includes more information than in the prior methods.
– The study did not include late response after the SIC test for the birch and grass allergens.
– An analysis of the impact of the severity of rhinitis would be interesting. However, the data on severity were not included in the present analysis and this data should be included in future similar studies.

## Introduction

Allergic diseases are prevalent in the working population, and the burden of work-related airborne pollen exposure might be substantial, especially among outdoor workers, resulting in work-related effects. Allergic rhinitis caused by or exacerbated by occupational pollen exposure is described in the literature, but the documentation is limited (1, 2). Both the health effects and the economic costs depend strongly on the combined total exposure and severity of the symptoms. One of the factors affecting this is “the priming effect,” a mechanism where repeated exposures to an antigen induce increased responses at similar exposure levels. For pollen allergy, the priming effect was first demonstrated for nasal symptoms (3). Later studies on the priming effect have included both the experimental challenges of repeated doses (4, 5) and the effect of the natural season (6, 7), exploring, for example, the symptom scores (8, 9), the nasal cells, and the bronchial response (10).

Remarkably there are, to the knowledge of the author, no recent studies published specifically on the seasonal priming effect, despite the inconsistency in the previous findings, and that further studies are warranted.

Occupational rhinitis is mentioned by the European Academy of Allergy & Clinical Immunology (EAACI) task force as “a disease of emerging relevance, which has received less attention compared with occupational asthma (11).” Generally, the majority of the studies on the bronchial response are performed on persons suffering from asthma. In the current study, we, therefore, chose to examine the bronchial response in the participants with allergic rhinitis, with no or only mild bronchial symptoms during the pollen season. According to the theory of “one airway, one disease” and the “united airway” (12, 13), these patients could also be expected to have increased bronchial response due to priming. However, this has not been explored in the previous studies.

The majority of studies on bronchial response to allergen exposures, apply measurements of the allergen dose eliciting a drop of e.g., 20% in forced expired volume in the first second (FEV_1_), the PD_20_, as a proxy for the degree of responsiveness. PD_20_ is estimated by linear interpolation between the two doses eliciting a drop below and above 20% on a logarithmic dose-response curve, although the response pattern is rarely linear. In this study, we wanted to examine the use of a non-linear regression model, to fit the log-dose-response curve and apply this in the estimate of the PD_20_ value.

This human exposure study aimed to evaluate the priming effect of natural seasonal allergen exposure level on the bronchial response in allergic rhinitis participants with none or mild asthmatic symptoms. We hypothesize that bronchoconstriction during specific allergen challenges is larger at the end of the natural pollen season, compared with the outside pollen season. The effect on the severity of bronchoconstriction is evaluated by applying a novel non-linear regression model to estimate PD_20_ from the log-dose-response curves. Furthermore, we wanted to assess the association between the baseline characteristics of the participants and the size of PD_20_.

## Materials and Methods

### Pollen

The pollen levels are recorded by the consumer organization Asthma Allergy Denmark at two sites in Denmark; Viborg and Copenhagen and measured according to the recommendations by the European Aerobiological Society (10, 12, 13).

### Study Participants

The potential study participants with self-reported allergic rhinitis were recruited following an open invitation (a stand in the local cafeteria) among the students at Aarhus University, Denmark, and skin prick testing (SPT) was performed to confirm the sensitization. The SPT was performed for grass (*Phleum pratense*), birch (*Betula verrucose*), artemisia, horse, dog, cat, house dust mites (HDM; *Dermatophagoides pteronyssinus* and *Dermatophagoides farina*), and fungal spores (*Cladosporium herbarum* and *Alternaria alternaria*). In this study, 92 persons were initially tested, and 36 participants with a positive SPT > 3 mm to grass or birch were recruited. No statistically significant difference in the weal size was found between those included in the study and those who decided not to participate. Of the 36 included, 17 underwent specific inhalation challenges (SICs) with birch allergen, and the remaining 19 participants underwent SIC with grass allergen (Table 1; Appendix 1). All the participants exposed to birch allergen, also had a positive SPT toward the grass, however, as all “out-of-season” exposures were performed far before the onset of the grass pollen season, this will not affect the results. Two of the 19 participants exposed to grass, also had a positive SPT toward birch.

**Table 1:** The study participant characteristics, mean (SD).

	**Grass**	**Birch**
*N* participants	19	17
Gender	F 10	F 10
	M 9	M 7
Weight	F 68 (13)	F 70 (14)
	M 77 (8)	M 86 (16)
Height	F 169 (5)	F 169 (6)
	M 183 (8)	M 179 (6)
Age	24.2 (2.7)	24.4 (2.3)
Wheal size, SPT mm^*^	9.4 (3.2)	6.5 (2.5)
Number of positive SPT (min./max.)	3.1 (1/6)	5.9 (4/8)
% Predicted, Baseline FEV_1_	F 99 (14)	F 97 (12)
	M 86 (12)	M 96 (12)

*F, female; M, male*.

* *Wheal size of (skin prick testing) SPT for the allergen applied in the specific inhalation challenges (SICs) (grass or birch)*.

Twenty of the participants reported respiratory symptoms (cough, wheeze, or chest tightness) more than two times per year, and 10 reported mild asthma. Only seven of them experienced asthma symptoms more than two times per year and none of them used asthma medication. None of the participants had recent exposure to smoking or had any recent infections, and none had used antihistamines within 72 h prior to the SIC.

All the participants had an initial FEV_1_ higher than 70% of the predicted and underwent a methacholine bronchial challenge test with a maximum cumulated dose of 4.51 mg.

The study was conducted in compliance with the Helsinki Declaration, and the protocol, enrollment procedure, and written consent forms were approved by the Scientific Ethics Committee for Central Denmark Region (M-20090215). Informed consent was signed by all the participants. There was no patient or public involvement in this study.

### Study Design

**Figure 1:**
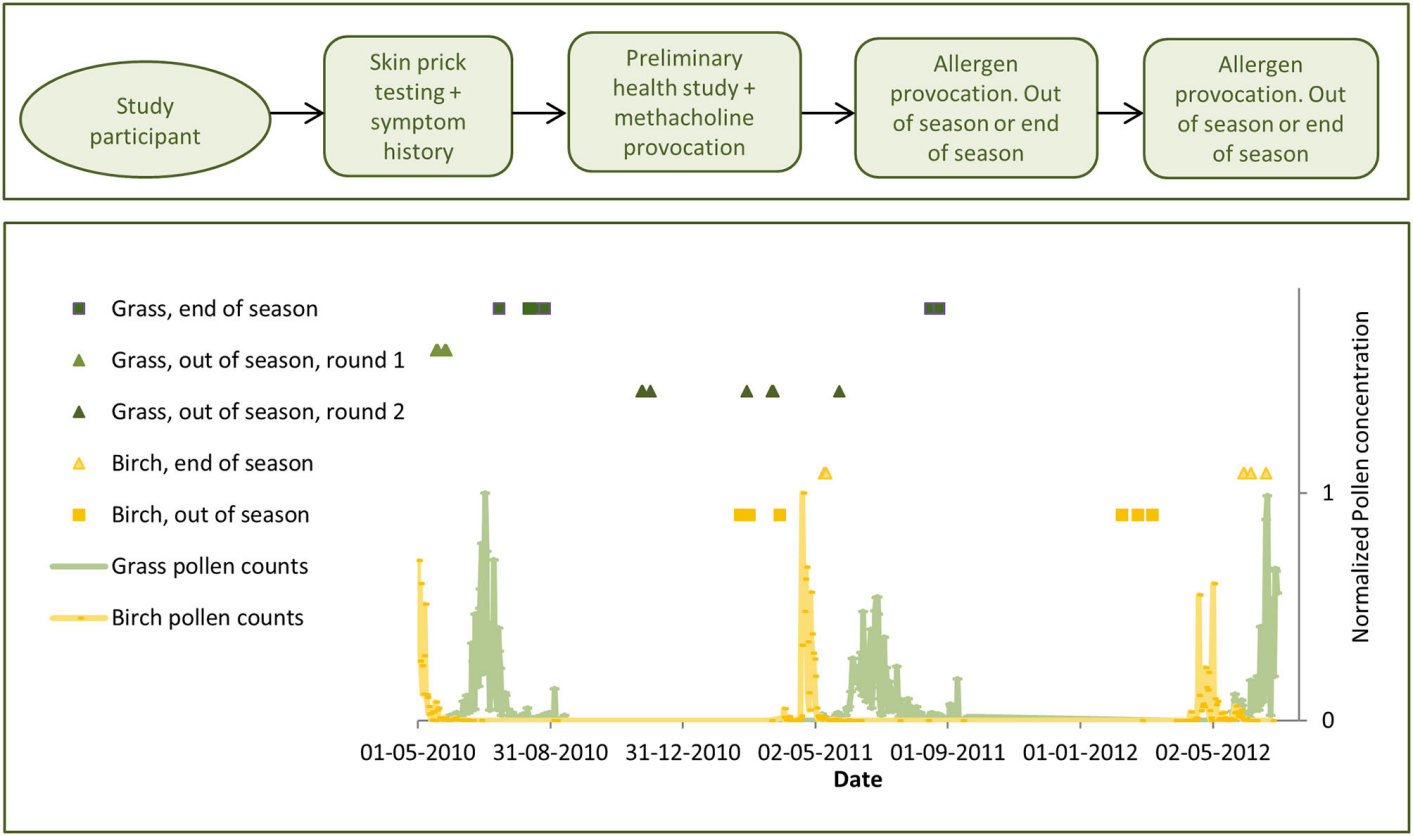
Study design (above) and timeline (below), depicting the specific inhalation challenges (SICs) and the daily pollen counts measured at the operational trap in the city of Viborg (below), normalized across the 3 years and two pollen species.

The study was conducted as a cross-over trial and performed during the four study days. During the first 2 days, the participants underwent SPT, symptom history, medical examinations, and methacholine challenge test. On the third and fourth day, SICs were performed in a human exposure chamber, either outside or at the end of the season for birch or grass pollen. For details, see Figure 1. The out-of-season measurements were collected in two rounds. Of the 19 participants who underwent SIC with grass allergen, 13 were included in a first round study, and six participants were included in a second round study outside the grass pollen season. All rounds of SIC were included in the analysis. “Outside season SICs” were performed outside the official grass or birch pollen season, defined by the registrations from the nearest operational pollen monitoring station in Viborg 60 km away (14–16). We did not have any information on the allergies to other tree pollens. However, none of the participants reported symptoms related to these pollen types and, therefore, no allergies from these were considered.

### Exposure Chamber

The majority of the SICs were conducted in a 79 m^2^ human exposure chamber (17, 18) at Aarhus University, Denmark, with 1–4 participants and the three investigators situated in the chamber. The 13 initial grass SICs out of season, were performed in a smaller 33 m^2^ chamber in conjunction with the large chamber, with participants seated in the large chamber between each SIC step. The participants were instructed to exhale in an extraction device to limit the exhalation of allergens into the chamber. Additionally, ventilation was installed in the exposure area. Airflow, temperature, humidity, and CO_2_ were measured every 60 s. The chambers were provided with filtered air at 22.5°C (SD 0.5°C) and humidity of 42.0% (SD 3.0%).

### Specific Inhalation Challenges

**Figure 2:**
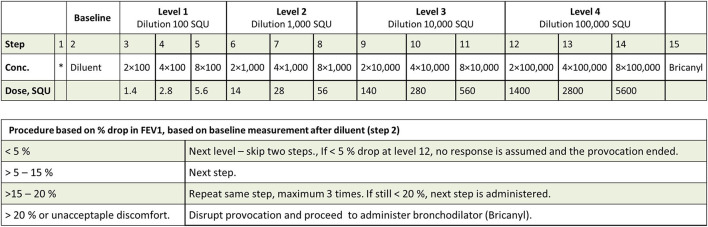
Protocol for the SICs. The administered dose is dependent on the magnitude of the drop in forced expired volume in the first second (FEV_1_) induced by the previous dose, resulting in either one step or one level increase in dose.

The allergen extracts were provided by ALK-Abelló (Hørsholm, Denmark) containing 100,000 SQ-U/ml of *P. pratense* (Phl p 5) or *B. verrucose* (Bet v 1). Solutions of increasing doses from 1.4 to 5,600 SQ-U were administered with a Spira Dosimeter nebulizer (Spira, Hämeenlinna, Finland) and FEV_1_ was measured with a handheld MicroDL spirometer (Micro Medical Limited, Rochester, UK) connected to a computer, where all data were logged. The SICs with aerosolized allergen were performed according to the procedure outlined in Figure 2. The doses were administered at 15 min intervals and considered to be cumulative as in the previous similar studies (19, 20). The total duration of the SICs were between 45 min and 2.5 h depending on the number of exposures required. FEV_1_ was measured 15 min after each inhalation as the best of three consecutive measurements. Baseline FEV_1_ was measured after inhalation of diluent. The applied protocol was developed to consider the prevention of exhaustion and, therefore, limiting the steps of the challenges.

After the SIC, the participants measured their FEV_1_ every 15 min for the first hour and thereafter hourly in the laboratory and later at home until bedtime for late phase responses, as a safety measure.

### Statistical Analysis

A natural log transformation of the administered dose of allergen concentrations was applied similar to other studies (4, 6, 7, 21, 22).

Bronchial hyperresponsiveness has been previously shown to depict a non-linear shape of the log-dose-response curves for the normal non-asthmatic participants (23, 24). We, therefore, fitted a non-linear regression model in the shape of a four-parameter logistic curve to the FEV_1_ data using the log cumulative dose as an independent variable:


$$\begin{array}{l} FEV1\left(x\right)=a+\;\frac{b-a}{1+\exp\frac{c-x}{d}} \end{array}$$

where *x* is the log cumulative dose, *a* is baseline FEV_1_, *b* is FEV_1_ at maximal log cumulative dose, *c* is the log cumulative dose corresponding to half the decrease in FEV_1_ (the inflection point), and *d* is a scaling parameter. The repeated measurements for each participant were taken into account by including random subject, and season (yes/no) within the subject effects, for each of the four parameters describing the FEV_1_-curve. The model validation was performed by inspecting the plots of observed and fitted FEV_1_-values against the cumulative dose and QQ residual plots.

Allergen responsiveness was defined as the natural logarithm to the cumulative dose of allergen theoretically eliciting a 20% drop in the baseline FEV_1_ (PD_20_). PD_20_ was estimated from the logistic model that included all the FEV_1_ observations, and not only the FEV_1_ responses to the last two doses administered, as in the previous studies.

The association between PD_20_ and the baseline characteristics of the study population was investigated using a linear regression model that included random subjects, and season (yes/no) within the subject effects, with PD_20_ on a logarithmic scale. The comparisons were performed using *post-hoc t*-tests based on the non-linear mixed effects regression analysis.

All the results were obtained using the nlme package in the R Programming Environment for Data Analysis and Graphics, Version 3.2.3 (25). A statistical significance was set at *p* = 0.05.

## Results

### Pollen Seasons

The natural seasonal pollen exposure during the study period was largely comparable with the 30-year average seasons in both the progress and magnitude. The grass pollen season in 2010 was marginally shorter and more intense than normal. In 2011, the birch pollen season was slightly less intense and ended slightly earlier than normal. Due to a data break in 2012, birch pollen was only recorded in Copenhagen. These indicated a normal season, although ending slightly abrupt due to cold weather in May. The yearly total grass pollen count for Viborg was 2,278 in 2010 and 2,097 in 2011. For birch pollen, the count was 3,585 in 2011 and not listed in 2012. This was lower than the counts for Copenhagen, 6,037 birch pollen. However, a nearby birch forest is leading to generally much higher counts at this site.

The normalized pollen concentrations are shown in Figure 1.

### Airway Responsiveness to Methacholine

A total of 44% of the subjects had a positive methacholine provocation, seven participants in each group. The modeled methacholine PD_20_ estimate for all the participants was 4.4 (95%-CI: 2.8; 6.8) mg methacholine bromide. No systematic errors in the residuals were seen.

### SIC for Grass and Birch Allergen

Eight of the 85 SICs were completed without the maximum dose administered or a 20% drop in FEV1 reached. No significant differences in the baseline FEV_1_ were seen between SICs outside the season and at the end of the season (birch, *p* = 0.98; grass, *p* = 0.91). Additionally, it was seen that 82% and 74% of the SICs at end of the season dropped more than 20% in FEV_1_ for birch or grass respectively; the numbers were 82% and 78% for SICs outside the seasons.

**Figure 3:**
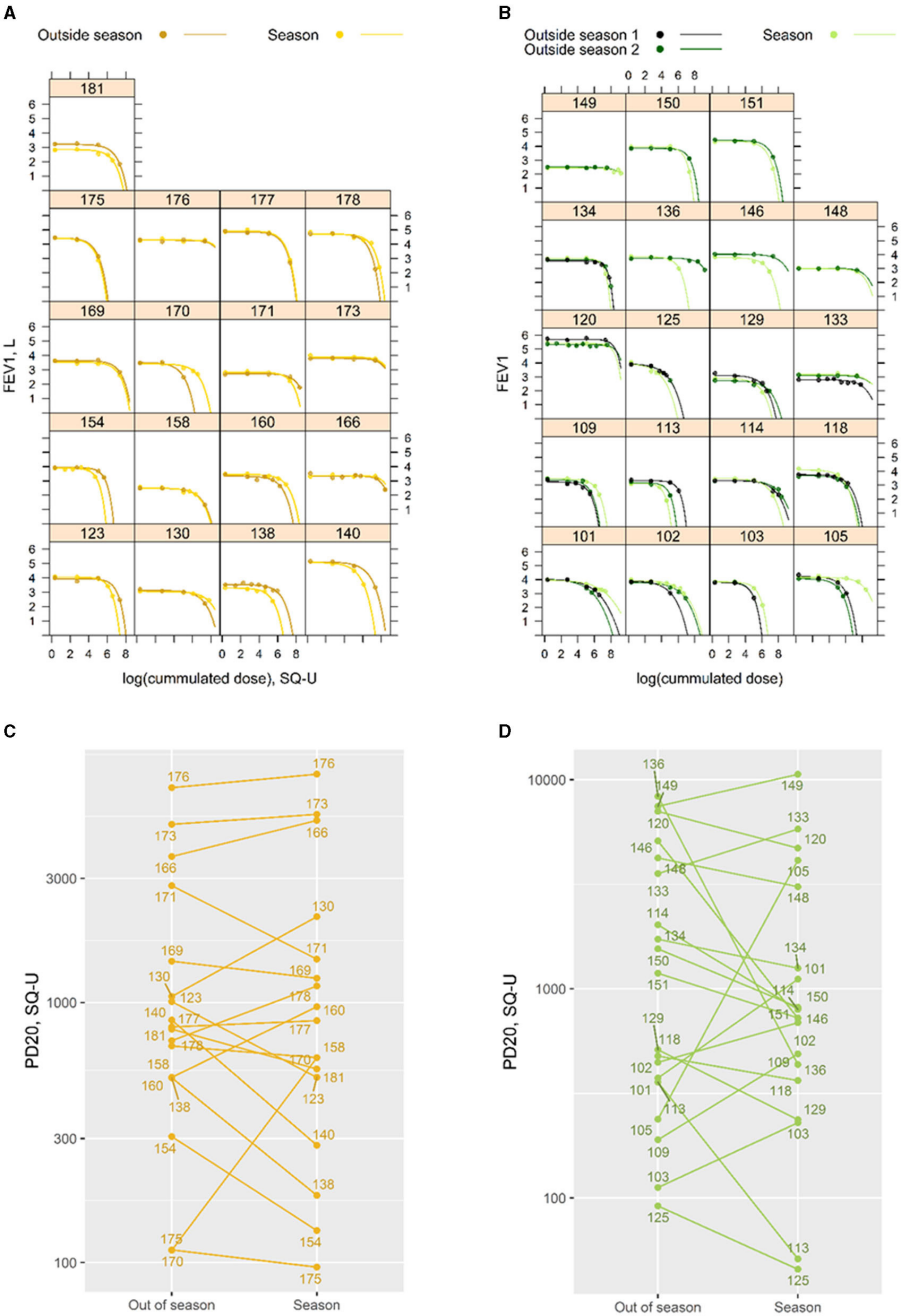
(**A** left + **B** right) Individual modeled dose-response profiles for the 17 participants challenged with birch allergen (left) and the 19 challenged with grass allergen (right). The numbers indicate the individual IDs of participants. Thirteen of the grass participants were challenged two times outside the season. (**C** left + **D** right) Individual PD_20_ estimates for challenges “out of season” and at the end of “season.” Estimates are shown for the 17 participants challenged with birch (left) and the 19 challenged with grass allergen (right). The numbers indicate the individual IDs of participants.

The four-parameter logistic model showed a good fit to the measured data (Figures 3A,B). No systematic errors in the residuals were seen. Individual PD_20_ estimates were modeled for all SICs, assuming both (1) no difference between exposure times (out of season and seasonal), and (2) allowing for a difference between the exposure times. No significant difference between the two models was found (birch, *p* = 0.30; grass, *p* = 0.39), indicating no significant influence of the seasonal priming effect.

Individual PD_20_ estimates for “out of season” and “season” SICs are shown in Figures 3C,D. For grass, 12 of the 19 participants required more allergen to drop 20% in FEV_1_ outside the season. For birch, this was true for nine of the 17 participants. The PD_20_ estimates (95% *CI*) based on all SICs for birch were 889 (494; 1599) SQ-U outside the season and 840 (464; 1519) SQ-U at the end of the season. For grass, PD_20_ estimates are 1020 (512; 2031) SQ-U outside the season and 792 (400; 1564) SQ-U at the end of the season. The PD_20_ estimate for the grass was associated with a 22% (−59; 48%) smaller dose at the end of the season for grass participants, and a 7% (−36; 36%) smaller dose by the end of the season for birch participants. However, the differences between season and outside the season were not statistically significant for either allergen (birch, *p* = 0.77; grass, *p* = 0.45).

**Figure 4:**
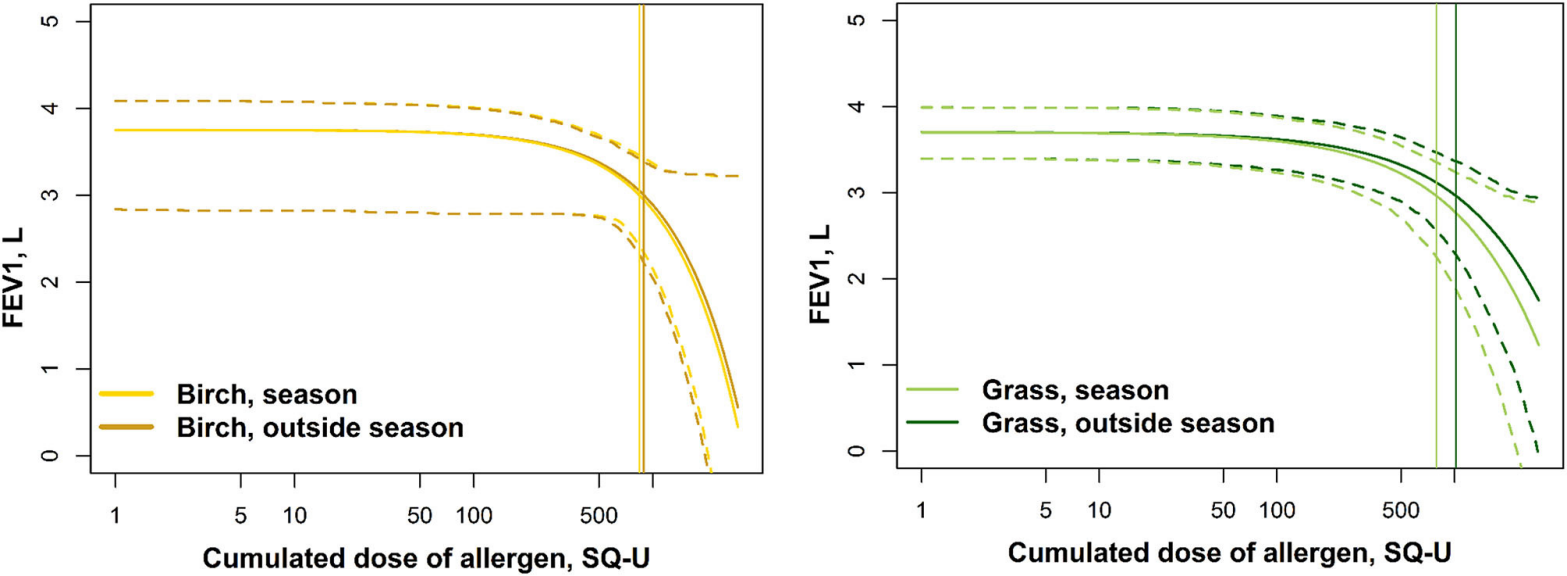
The modeled log-dose-response curves with 95% CI. The vertical lines indicate the modeled PD_20_ estimates.

The modeled dose-response curves (FEV_1_/log cumulated dose of allergen) are shown in Figure 4 with indications of the modeled PD_20_ estimates. No significant differences in the shape or magnitude of the curves were seen between the “out of season” and “season” SICs.

### Associations Between the Baseline Characteristics and PD_20_

As no statistically significant effect of season was seen on the PD_20_ estimates, the analysis on associations between the baseline characteristics (methacholine PD_20_, SPT, number of allergies, and baseline FEV_1_) of the participants and allergen PD_20_, was performed on a combined dataset that included both birch and grass allergens. Only the size of the SPT for participants with grass allergy showed a clear and significant association with allergen PD_20_. On average, a 1 mm larger reaction in the skin prick test was associated with a 26% lower PD_20_ (Table 2).

**Table 2:** The associations between the baseline characteristics for the two participants groups (birch and grass), and Allergen-PD_20_, Methacholine-PD_20_, size of SPT, number of positive SPT, and baseline forced expired volume in the first second (FEV_1_).

	**% Change in PD_**20**_**	***P***
Methacholine	% change in PD_20_ allergen per 10% change in PD_20_ methacholine	
Birch	3.75 (−1.15; 8.88)%	0.158
Grass	0.77 (−4.21; 6.02)%	0.770
Skin prick test	% change in PD_20_ per mm change in SPT-wheal	
Birch	−17.95 (−33.47; 1.18)%	0.085
Grass	–*25.78 (−36.64; −13.10)%*	0.002^*^
Number of pos. SPT	% change in PD_20_ per pos. SPT	
Birch	−19.39 (−45.70; 19.67)%	0.304
Grass	27.77 (−11.32; 84.21)%	0.208
Baseline FEV_1_	% change in PD_20_/(l/sec) change in Baseline FEV1	
Birch	−17.60 (−59.99; 70.80)%	0.608
Grass	5.05 (−58.83; 172.82)%	0.920

* *Statistical significant*.

## Discussion

In this study, we could not confirm a priming effect of neither grass nor birch pollen, the dominant pollen exposures for outdoor occupations, among the persons with allergic rhinitis and no or mild asthma. This was true for both the shape and the magnitude of the modeled dose-response curves, as well as for PD_20_ for both the grass and birch allergens. Among the baseline characteristics, only the size of the skin prick test for grass allergen was significantly positively associated with PD_20_. The model depicted a good fit for the data.

Although only seven of the 36 participants reported more than the mild asthmatic symptoms a few times a year, ~80% of the SICs resulted in a reduction of more than 20% in FEV_1_. This is consistent with the theory of the “United airway,” and our assumption of an allergic bronchial response in the rhinitis participants. The modeled individual PD_20_ estimates were lower by the end of the season for 12 of the 19 grass participants, and the overall PD_20_ estimate was associated with a 24% decline in dose. For birch, nine of the 17 participants had a lower PD_20_ by the end of the season, and the overall PD_20_ estimate was associated with a 6% smaller dose. Although none of the differences were statistically significant, the results for the grass participants indicated a lower tolerance by the end of the season, and a potential priming effect of the seasonal exposure.

The wide 95%-CI intervals indicate that future studies should include a larger number of participants and/or more homogeneous experimental settings to reduce the effect of random variation.

The differences in the effect of pre-priming induced by homologous pollen types (26), could also serve as an explanation for the tendency for a difference between the response in grass and birch pollen SICs. This could be an issue for those suffering from birch allergies since many of the early spring flowering trees have homologous allergens and often induce cross-reactivity. In this study, we did not have SPT or IgE results for any of these allergens. Although none of the participants report symptoms related to these tree pollen, pre-priming could still occur if sensitization is present, since the exposure to low doses of the allergen, not eliciting symptoms, may still cause bronchial inflammation and increase responsiveness (5, 27). However, this is not expected to impact the results, and the effects can be assumed to be minor.

The natural priming exposure of birch pollen may have been blunted in 2012, when the concentrations fell slightly more abruptly than normal, due to cold weather. This resulted in difficulties in planning in relation to the pollen season, and two of the “end of season SICs” with birch allergen in 2012 were not performed until 3 weeks after the season. The effect of priming by repeated exposures has previously been seen to almost vanish 3–6 weeks after the exposures (5), and therefore, we cannot be sure that the two late challenges were affected by the seasonal priming. Another issue related to the timing was one out-of-season SIC performed shortly before the start of the season, and this person is, therefore, at risk of having been pre-primed by the low pre-season concentrations. This is especially a risk for birch pollen, where long distance transported pollen, may arrive before the local season starts (28). Two of the participants challenged with grass allergen also had a positive SPT to birch and may have been pre-primed by the seasonal exposure to birch. The lowest PD_20_ observed for the out of season round one, maybe due to this pre-priming by the birch season. Excluding these SICs that did, however, not alter the results, and pre-priming is, therefore, not considered to have affected the results.

Both the rate and degree of symptoms could be affected by the non-specific pre-priming in participants co-sensitized to the perennial allergies (29, 30). Fourteen birch participants and seven grass participants in this study were also sensitized to HDM. Only half of the participants with positive SPT to HDM experienced symptoms from this, however, sensitization alone could still affect the pre-priming. An assessment of the differences in individual PD_20_-estimates for grass participants out-of-season challenges showed a 3-fold increase of the PD_20_-estimates for the seven participants with HDM sensitization, compared with the 12 without (*p* = 0.06), as well as 15 times larger difference in PD_20_ between the season and out-of-season. This strongly indicates that there was no separate priming effect of this sensitization on the bronchial response in this study.

The applied protocol was developed to consider the prevention of exhaustion and, therefore, limiting the steps of the challenges. This resulted in 8 SICs completed without the maximum dose administered or a 20% drop in FEV_1_ reached. Although most of the participants experienced a 20% or greater drop in FEV_1_ during the SICs and many of the remaining were non-respondent at all challenges, some may also have a response if the maximum dose had been administered.

The model demonstrated a good fit to data and a suitable method for producing the PD_20_ estimates and the modeling log dose-response curves. This method allows for all information on the dose-response pattern to be included in the calculation of the PD_20_-estimate, and not solely on the individual response to the last two doses administered, as in the previous studies. Although the individual differences were apparent, the modeled dose-response curve based on all the SICs indicated similar response patterns for both the allergens and exposure times. To the knowledge of the author, this has not been shown previously. We believe that the method shows great potential, however, it should be replicated in other similar studies before conclusions can be made.

The previous studies examining the effect of seasonal exposure on the bronchial response of allergens are heterogeneous in methods, measures of effects as well as in results, and the results are inconsistent. Dente et al. (7) found a significant 3-fold decrease in PD_20_ for asthmatics in the grass pollen season compared with the outside season, but only for those having a dual response, i.e., both early airway response (EAR) and late airway response (LAR). Paggiaro et al. (22) also found a 3-fold decreased PD_15_ during the grass pollen season in the asthmatic participants, compared with the outside the season, but only for those who shifted from an EAR+LAR response outside the season to an EAR within the season. No consistent measurements of LAR were conducted in the current study, where the focus was on EAR, however, future studies should also include this measure. From the theory of “one airway, one disease” and the “United airway” (12, 13), it was our hypothesis, that the participants with allergic rhinitis symptoms, would also show an increased bronchial response to allergen due to priming. Even though we did see a tendency to a priming effect among the grass-sensitized participants, the results were far from statistically significant.

The priming effect has been expressed previously in the terms of increased non-specific bronchial hyper-responsiveness for both the seasonal and repeated administered allergen doses for different subgroups of study populations. De Bruin-Weller et al. (4) found a significant priming effect, but only for those having high non-specific bronchial hyper-responsiveness. Ihre and Zetterstom (5) found a priming effect for the subgroup with a high IgE level, and Dente et al. (7) found the increased bronchial response to methacholine, for those with LAR outside the pollen season. All these studies were, however, performed on the asthmatic participants. There are some studies, which find a general increased non-specific bronchial hyperresponsiveness during the season in their entire study population, and also in the mixed groups, such as non-asthmatics. A study by Crimi et al. (6) found a significant increase in both EAR and LAR following natural seasonal exposure for all the participating patients with inhalant allergy by birch pollen and suggests a correlation with increasing IgE as a part of the mechanism, and Walker et al. (31) found a significant decrease in methacholine PD_20_ during the pollen season for all the tested patients. Madonini et al. (32) found significantly lower carbachol-PD_20_ in the season than the outside season in 27 grass atopic participants without asthma, and generally a larger priming effect in those who had a positive response outside season. All the studies were performed on the participants with no HDM sensitization and, therefore, no risk of pre-priming.

The size of SPT for grass sensitization was the only baseline characteristic associated with PD_20._ We did not find a similar association for birch SPT and this may be due to the on average smaller wheal sizes for this allergen and, therefore, less variability in the measurements. These findings are consistent with the previous studies (22, 33).

We expected to find an association between allergen PD_20_ and baseline unspecific bronchial responsiveness to methacholine. A study by De Bruin-Weller et al. (4) found significantly higher non-specific bronchial responsiveness following allergen exposure, and a significant correlation of this with baseline non-specific bronchial responsiveness, but only in the participants with high baseline histamine PD_20_. This could indicate that the association may be blunted for those who are strongly responsive. Another study by Barnig et al. (34) also found an association between the grass allergen PD_20_ and methacholine reactivity. The lack of such a finding in our study may be due to the fact that only 44% of our participants had a positive methacholine provocation test.

Despite pollen exposure is greatly increased for those with outdoor occupations, only few studies on occupational pollen exposure and rhinitis are available. The current results do not support that the workers are more prone to severe symptoms late compared with early in the pollen season. For bell pepper pollen, the rhinitis symptoms mostly develop within the first 2–3 years after exposure onset (2). However, as pollen exposure is high in the environment in general and not only within the occupational setting, the occupational contribution can be difficult to disentangle. It is also important to note that this study was performed among university students and that the average outdoor worker can be assumed to be both older and from a lower socioeconomic class. This could potentially give a difference in the overall health condition. Furthermore, it can be speculated, the outdoor occupation would not be chosen, by those suffering from severe and prolonged pollen allergy. Therefore, our results may not directly be representative of the outdoor workers.

## Conclusion

In this study, we could not confirm a priming effect of neither grass nor birch pollen among the individuals with allergic rhinitis and no or mild asthma although we did see a tendency to lower PD_20_ by the end of the season among the participants with allergic rhinitis. The size of the SPT for grass at baseline was inversely associated with allergen PD_20_. The modeled dose-response profiles showed a good fit for data.

The current study presents a strong method for determining PD_20_ estimates. Further studies are needed to replicate the results and present representative estimates for other (age and socioeconomic) groups with allergic rhinitis and only mild or no allergic asthma, a large and previously not frequently studied patient group. The study confirms that the priming effect is not an important factor for the magnitude of the allergic reaction in all the patients. However, the study was not powered to analyze the impact of different endotypes and did not systematically evaluate LAR. In future studies, the priming effect of seasonal exposure should focus on the impact of IgE levels, response pattern, and the level of non-specific bronchial responsiveness.

## Data Availability Statement

The raw data supporting the conclusions of this article will be made available by the authors, without undue reservation.

## Ethics Statement

The studies involving human participants were reviewed and approved by the Scientific Ethics Committee for the Central Denmark Region (M-20090215). The participants provided their written informed consent to participate in this study.

## Author Contributions

BB mainly contributed to the statistical analysis and PR to the experimental setup. PØ, JB, and VS performed the data analysis and wrote the first manuscript draft. All the authors have provided substantive contributions in study design, data collection, interpretation of results, revising the article critically for important intellectual content, and final approval of the version to be published.

## Funding

This study was fully funded by Aarhus University, Denmark. PØ was supported by BERTHA—the Danish Big Data Centre for Environment and Health funded by the Novo Nordisk Foundation Challenge Programme (grant NNF17OC0027864) during the completion of the manuscript.

## Conflict of Interest

The authors declare that the research was conducted in the absence of any commercial or financial relationships that could be construed as a potential conflict of interest. The handling editor declared a shared affiliation, though no other collaboration with the authors at the time of the review.

## Publisher's Note

All claims expressed in this article are solely those of the authors and do not necessarily represent those of their affiliated organizations, or those of the publisher, the editors and the reviewers. Any product that may be evaluated in this article, or claim that may be made by its manufacturer, is not guaranteed or endorsed by the publisher.

## Appendix 1

**Table A1:** Sensitization profiles for all study subjects.

**ID**	**Grass**	**Mugwort**	**Birch**	**Horse**	**Dog**	**Cat**	**D. farinae**	**D. ptero**.	**Alternaria**	**Clados**.	**Aspergi**.
Birch 1	6	5	3,5	*	*	*	7	7	*	*	*
Birch 2	6	*	14	4	4	*	5	5	8	*	*
Birch 3	7	7	6	*	2	5	*	*	*	*	*
Birch 4	7	*	7	*	4	5	*	9	*	*	*
Birch 5	7	*	7	*	6	9	5	11	*	*	*
Birch 6	9	4	7	*	5.5	*	*	4.5	3	*	*
Birch 7	10	3	4	*	4	*	3	5	2	*	*
Birch 8	10	8	7	*	5	5	*	*	*	*	*
Birch 9	11	10	8	*	8	11	6	7	5	*	*
Birch 10	11	7	5	5	5	5	5	8	*	*	*
Birch 11	12	*	5	*	4	*	8	9	*	4	4
Birch 12	12	*	5	*	5	17	6	6	*	*	*
Birch 13	13	11	6	*	*	*	6	6.5	*	*	*
Birch 14	15	*	4	*	3	*	5	8	*	*	*
Birch 15	16	*	5	*	5	3	2	5	*	*	*
Birch 16	20	4	9	*	4	*	*	*	*	*	*
Birch 17	32	*	8	3	7	9	7	26	7	*	*
Grass 1	4	*	*	*	*	*	*	*	*	*	*
Grass 2	5	*	*	*****	3	*	*	3	*	*	*
Grass 3	5	*	3	*	*	*	4	9	*	*	*
Grass 4	6	7	*	*****	*	*	*	*	*	*	*
Grass 5	7	5	*	*	4	*	*	*	*	*	*
Grass 6	8	*	*	*	4	*	3	*	*	*	*
Grass 7	8	5	*	*	5	8	4	8	*	*	*
Grass 8	8	12	*	*	3	7	4	7	*	*	*
Grass 9	9	4	*	*****	5	*	*	*	*	*	*
Grass 10	9	*	*	*	*	*	*	*	*	*	*
Grass 11	10	5	*	*	5	*	*	7	*	*	*
Grass 12	11	5	4	*****	5	10	*	*	*	*	*
Grass 13	11	*	*	*****	*	*	*	*	*	*	*
Grass 14	11	*	*	*****	4	*	*	*	*	*	*
Grass 15	11	*	*	*	*	*	*	*	*	*	*
Grass 16	13	*	*	*	*	*	*	*	*	*	*
Grass 17	13	*	*	*	5	*	*	*	*	*	*
Grass 18	14	*	2	*	5	4	*	*	*	*	*
Grass 19	15	4	*	*****	5	12	6	10	*	*	*

*SPT size in mm, *no response*.
